# Upregulation of PD-1 expression on circulating CD8+ but not CD4+ T cells is associated with tuberculosis infection in health care workers

**DOI:** 10.1186/s12865-021-00433-9

**Published:** 2021-06-25

**Authors:** Cui-lin Shi, Jian-ping Zhang, Ping Xu, Jin Li, Jie Shen, Mei-ying Wu, Zhi-jian Ye, Xin Yu, Hua-feng Song, Hui Chen, Jun-chi Xu, Yu Pang, Jian-an Huang

**Affiliations:** 1 Department of Pulmonary and Critical Care Medicine, The First Affiliated Hospital of Soohow University, Suzhou, 215006 Jiangsu Province China; 2 The Fifth People’s Hospital of Suzhou (The Affiliated Infectious Diseases Hospital of Soochow University), 215131, Suzhou, Jiangsu Province China; 3 Department of Bacteriology and Immunology, Beijing Chest Hospital, Capital Medical University, Beijing Tuberculosis and Thoracic Tumor Institute, Beijing, 101149 Beijing China

**Keywords:** Health care worker, Latent tuberculosis infection, PD-1, Immunodepletion, T cell

## Abstract

**Background:**

Health care workers (HCWs) are at risk for occupationally acquired *Mycobacterium tuberculosis* infection and tuberculosis (TB) disease due to repeated exposure to workplace tubercle bacilli. To determine whether continual mycobacterial stimulation correlates with increased expression of inhibitory T cell receptors, here we compared PD-1 receptor expression on surfaces of circulating T cells between naïve (uninfected) HCWs and HCWs with latent TB infection (LTBI).

**Result:**

Data collected from 133 medical workers who met study selection criteria were included in the final analysis. QuantiFERON-TB Gold In-​Tube (QFT-GIT) testing yielded positive results for 32 HCWs, for an overall LTBI rate of 24.1%. Multivariate analysis identified HCW length of service > 15 years as an independent risk factor for a positive QFT-GIT result. In addition, comparisons of blood T cell subgroup profiles between QFT- and QFT+ groups indicated QFT+ subjects possessed greater proportions of mature (TM), transitional memory (TTM) and effector memory (TEM) CD4+ T cell subgroups and lower proportions of naïve T cells (TN). Moreover, the QFT+ group percentage of CD8+ T cells with detectable surface PD-1 was significantly higher than the corresponding percentage for the QFT- group. Meanwhile, no statistical intergroup difference was observed in percentages of CD4+ T cells with detectible surface PD-1.

**Conclusions:**

Our data demonstrated that upregulated PD-1 expression on circulating CD8+, but not CD4+ T cells, was associated with latent TB infection of HCWs. As compared to other hospitals, occupational TB infection risk in our hospital was substantially mitigated by implementation of multitiered infection control measures.

**Supplementary Information:**

The online version contains supplementary material available at 10.1186/s12865-021-00433-9.

## Introduction

Tuberculosis (TB), caused by *Mycobacterium tuberculosis* complex, has great significance for public health worldwide, with an estimated one-third of the world’s population harboring latent TB infection (LTBI) [1, 2]. Importantly, LTBI serves as the source of all new incident cases of active TB disease, highlighting the fact that the target goal of eliminating TB worldwide by 2035 cannot be achieved without addressing LTBI in populations [3, 4]. In order to prevent LTBI, aggressive strategies are necessary to halt LTBI disease progression to active TB in order to break the cycle of transmission that continues to fuel the current worldwide TB epidemic. Nevertheless, implementation of such strategies alone will be insufficient for eradicating TB, since continually increasing LTBI burden in vulnerable populations must also be addressed [4]. Ultimately, a better understanding of the pathogenesis of latent TB infection would greatly facilitate development of targeted interventions to recognize and protect naïve individuals from TB infection.

As a significant occupational health problem, health care workers (HCWs) are at high risk of TB exposure [5, 6]. In a recent systematic review, HCWs in low- and middle-income countries continue to possess unacceptably high TB infection incidence and prevalence rates, with prevalence ranging from 9 to 86% [7]. Previously reported independent risk factors for LTBI have included a greater number of years of service, work location and TB patient contact [8–10]. Although the World Health Organization (WHO) has endorsed implementation of infection control measures to protect HCWs, a highly vulnerable group [11], we believe that greater emphasis should be placed on discovering novel biomarkers with improved performance and predictive value toward monitoring and reducing HCW TB infection risk.

In healthy adults, properly functioning innate and adaptive immune systems are vital for controlling and eradicating *M. tuberculosis* infection [12]. As part of the human adaptive immune system, T lymphocyte-mediated cellular immunity is central to the protective *M. tuberculosis* response [13]. Under conditions of chronic antigen stimulation, such as persistent infection and cancer, T cell effector functions can be dampened by activities of T cell inhibitory receptors, a state termed “exhaustion” [14]. HCWs are at particularly high risk for infection with occupationally acquired *M. tuberculosis* that can progress to active TB disease after persistent exposure to tubercle bacilli [2]. The question has been raised as to whether such continuous mycobacterial stimulation in health care settings correlates with increased expression of inhibitory receptors on T cells that supports high HCW TB infection rates. In this study, we compared PD-1 expression on circulating T cells obtained from naïve (uninfected) HCWs and HCWs latently infected with *M. tuberculosis*. Our objective was to elucidate the role of T cell exhaustion in TB infection to gain valuable insights to guide the development of strategies to halt the unrelenting spread of tuberculosis.

## Materials and methods

### Subjects

**Fig. 1:**
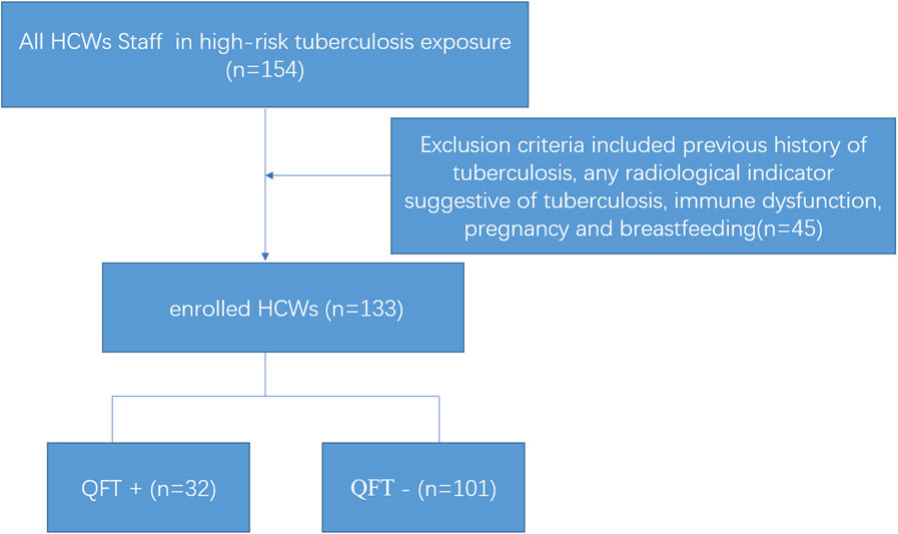
Study flow diagram. HCWs: health care workers; QFT: QuantiFERON-Gold In-Tube assay

Research related to use of human subjects as was conducted in this study complied with all relevant national regulations, institutional policies and tenets of the Helsinki Declaration. The design of this study has been approved by the Ethics Committee of the Fifth People’s Hospital of Suzhou. Informed consent was obtained from all participants prior to initiation of the study. We recruited medical HCWs from the Fifth People’s Hospital of Suzhou who engaged in high-risk duties involving exposure to TB patients. Study subjects included clinical physicians, nurses, imaging department workers and laboratory workers. A total of 153 HCWs were initially screened in our study. Exclusion criteria included previous history of tuberculosis, any radiological indicator suggestive of tuberculosis, immune dysfunction, pregnancy and breastfeeding. Ultimately, 133 HCWs who met study selection criteria were included in our final analysis (Fig. 1). Of these, 32 HCWs in the QFT+ group were successfully matched according to sex and age to 31 subjects in the QFT- group to create 31 clinical QFT+/QFT- subject pairs who provided blood for flow cytometric analyses.

### QuantiFERON-gold in-tube (QIF-GIT) assay

Blood samples were obtained after subjects fasted overnight through early morning. For each subject, three heparin tubes were collected: (1) a negative-control tube (NIL tube), (2) an antigen tube (AG tube) containing a coating of specific *Mycobacterium tuberculosis* antigens (ESAT-6, CFP-10, TB 7.7) that came into contact with the subject’s T cells in the blood sample) and (3) a positive-control tube containing phytohemagglutinin-P (PHA) (MIT tube). The concentration of IFN-γ secreted by T cells was measured by ELISA. The results were measured in IU/ml and interpreted in accordance with the manufacturer’s recommendations as negative, positive or indeterminate [15].

### Flow cytometric analysis

Fluorochrome-labeled monoclonal antibodies CD45RA, CD45RO, CCR7, CD4, CD8 and HLA-DR were purchased from BD Pharmingen (San Diego, CA, USA) and used for flow cytometry based on a gating strategy as previously reported [16]. For each test, 50 μL of fresh heparinized whole blood of test subjects or healthy donors was incubated with indicated antibodies (10 μL) for 15 min then lysed with FACSTM lysing solution (BD Biosciences, San Jose, CA, USA). Samples were subsequently washed with phosphate buffered saline, fixed then subjected to flow cytometric analysis using a BD FACSAria system with BD FACSDiva software.

### Statistical analysis

All data were analyzed using Graph Pad Prism 5.0 software and SPSS 22.0 and were presented as means ± the standard deviations (SDs). Mann-Whitney U-test and Student’s t-test were used for the continuous variables such as mean of the age, and chi-square test was used to compare the categorical variables. Associations between risk factors and QFT results were evaluated using multivariate logistic regression analysis; a two-tailed *p* < 0.05 was considered statistically significant.

## Results

### Latent TB infection in HCWs

A total of 133 HCWs were included in our analysis. Baseline characteristics and absolute numbers of HCW subjects are shown in Table 1. The majority of HCWs were female (*n* = 105, 78.9%). The HCW median age was 35.1 years and the median number of years of service was 12.5 years. More than 59.4% of participants were nurses working in TB facilities. Diabetes and hypertension were noted in 4 (3.0%) and 5 (3.8%) participants, respectively.

**Table 1:** Baseline Characteristics of medical worker

Characteristics	Total	QFT +(***n*** = 32)	QFT -(***n*** = 101)	Statistical value	***P*** value
Sex					
Male	28 (21.1)	5 (15.6)	23 (22.8)	0.747	0.387
Female	105 (78.9)	27 (84.4)	78 (77.2)		
Age	35.1 ± 8.4	38.0 ± 8.0	34.2 ± 5.2	−1.763	0.078
Working time (year)	12.5 ± 10.0	16.5 ± 8.5	11.2 ± 6.2	−2.833	0.005
< =2	15 (11.3)	1 (3.1)	14 (13.9)	13.067	< 0.001
(3–5)	20 (15.0)	0	20 (19.8)		
(6–15)	61 (45.9)	16 (50)	45 (44.6)		
(16–25)	12 (9.0)	6 (18.8)	6 (5.9)		
> 25	25 (18.8)	9 (28.1)	16 (15.8)		
Occupation					
Doctor	34 (25.6)	11 (34.3)	23 (22.8)	7.891	0.048
Nurse	79 (59.4)	21 (65.7)	58 (57.4)		
Laboratory staff	13 (9.8)	0	13 (12.8)		
Radiologist	7 (5.3)	0	7 (7.0)		
Chronic medical illness					
Hypertension	4 (3.0)	2 (6.3)	2 (2.0)	3.058	0.09
Type 2 diabetes	5 (3.8)	3 (9.4)	2 (2.0)	1.3	0.244
Clinical biochemical index					
White blood cell count(WBC), 10^9/L		6.063 ± 0.938	6.701 ± 1.651	−0.993	0.321
Lymphocyte count(L), 10^9/L		2.059 ± 0.451	2.131 ± 0.421	−0.704	0.481
Alanine aminotransferase(ALT), U/L		17.969 ± 9.469	21.457 ± 12.707	−0.993	0.321
Plasma creatinine, μmol/L		59.941 ± 8.941	63.823 ± 8.823	−1.752	0.08
Cellular immunological index					
CD4^+^ T cell count, 10^^9^/L		0.727 ± 0.246	0.763 ± 0.225	0.772	0.442
CD8^+^ T cell count, 10^^9^/L		0.569 ± 0.193	0.558 ± 0.213	−0.263	0.793
T cell count, 10^^9^/L		1.391 ± 0.283	1.431 ± 0.315	−0.156	0.876
B cell count, 10^^9^/L		0.242 ± 0.097	0.248 ± 0.082	−0.024	0.981
NK cell count, 10^^9^/L		17.061 ± 5.231	18.14 ± 6.205	−0.303	0.762

### Risk factors for latent TB infection in HCWs

We further analyzed risk factors for latent TB infection in HCWs in our cohort. Among the HCWs, blood of 101 subjects tested negative by QFT-GIT, while the other 32 subjects had positive QFT-GIT results, for an overall HCW LTBI rate of 24.1%. Results of analysis of relationships among demographic and clinical characteristics and QFT-GIT responses are summarized in Table 1. The highest LGBI prevalence rate was found in nurses (21/133, 65.7%) and the second-highest rate as found in doctors (11/133, 34.3%). Notably, no laboratory staff or radiologists tested positive via QFT-GIT, while a high number of years of practice was significantly associated with increased TB risk. By contrast, no correlation was found between LTBI status and differential white blood cell count. Meanwhile, multivariate analysis revealed that independent risk factors for positive QFT-GIT results included number of years of service as a HCW of > 15 years [adjusted odds ratio (aOR): 0.249, 95% confidence interval (95% CI): 0.090–0.686, *P* = 0.007) (Table 2).

**Table 2:** Univariate and multivariate analysis of factors associated with positive or negative QFT-G result

Variable	Univariate			Multivariate		
	OR	95%CI	***P***	0R	95%CI	***P***
Age	1.053	1.005–1.103	0.029			
Sex	1.592	0.551–4.603	0.390	0.827	0.172–3.973	0.813
work time (≤15 vs > 15)	3.168	1.368–7.338	0.007	0.249	0.090–0.686	0.007
Hypertension	0.303	0.041–2.244	0.242	0.564	0.062–5.097	0.61
Plasma creatinine, μmol/L	0.975	0.942–1.009	0.146	0.969	0.923–1.017	0.203
CD4^+^T cell count, 10^^9^/L	0.997	0.937–1.061	0.928	0.838	0.002–479.941	0.956
CD8^+^ T cell count, 10^^9^/L	1.038	0.981–1.099	0.198	23.342	0.031–17,527.830	0.351
T cell count, 10^^9^/L	0.784	0.286–2.153	0.637	0.810	0.004–157.836	0.937
B cell count, 10^^9^/L	0.597	0.016–21.626	0.778	0.404	0.001–136.131	0.76
NK cell count, 10^^9^/L	0.981	0.928–1.037	0.495	0.452	0.028–7.207	0.574

### Subpopulations of human memory T cells

**Fig. 2:**
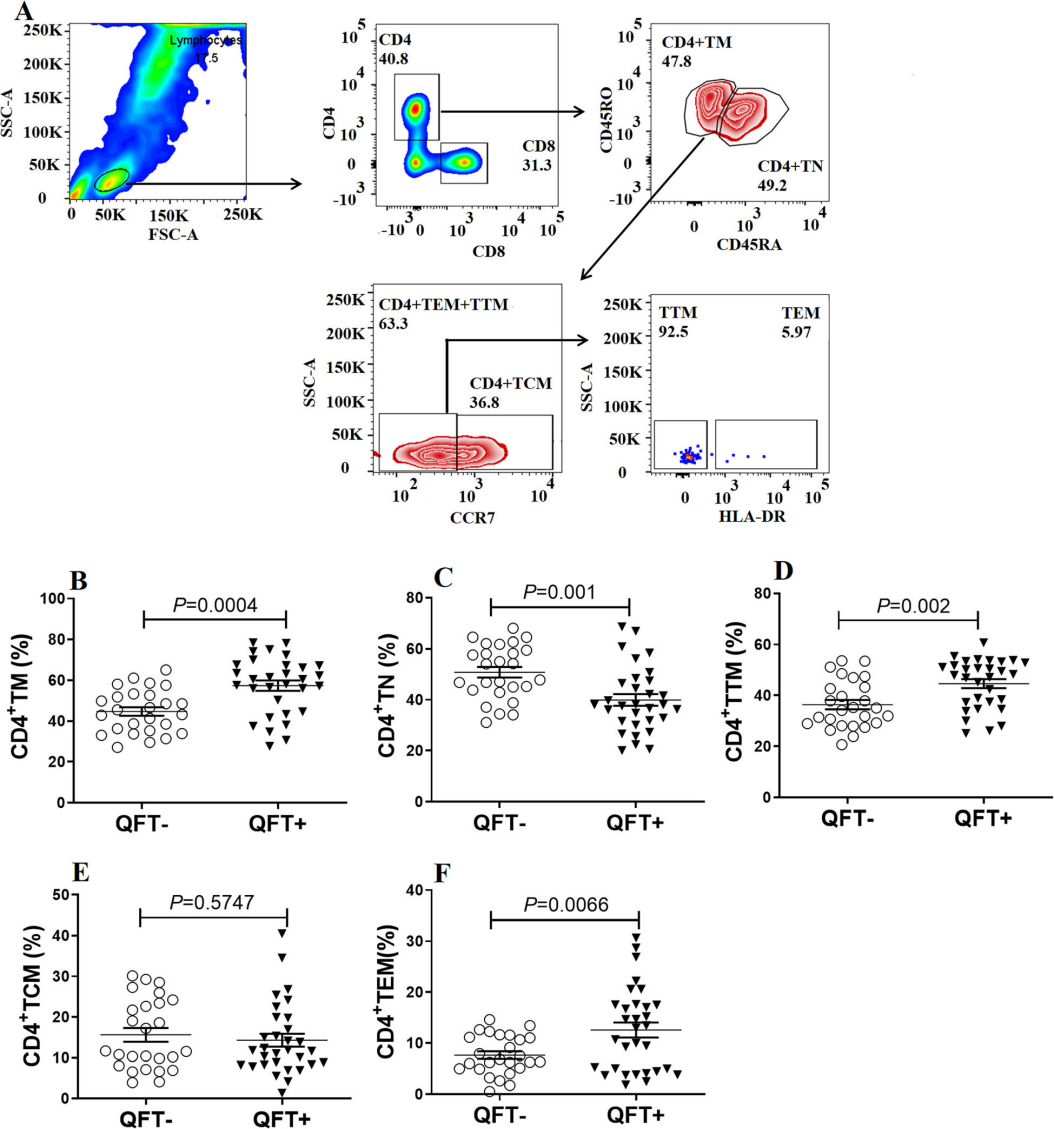
Gating strategy and expression profiles of memory CD4+ T cell subpopulation in latent TB-infected HCWs. **A** Gating strategies and representative results for the CD4+ T cell subpopulation. **B** Analysis of expression differences between memory CD4+ T cells of QFT+ and QFT- groups. **C** Analysis of expression differences between naïve CD4+ T cells in QFT+ and QFT- groups. **D** Analysis of expression differences between transitional memory CD4+ T cells in QFT+ and QFT- groups. **E** Analysis of expression differences between central memory CD4+ T cells in QFT+ and QFT- groups. **F**Analysis of expression differences between effector memory CD4+ T cells in QFT+ and QFT- groups

Based on expression of lymphocyte surface biomarkers, human memory T cells can be categorized according to differentiation stage into mature (TM), naïve (TN), central memory (TCM), transitional memory (TTM), and effector memory (TEM) T cells [17]. We observed that blood of subjects of the QFT+ group contained an overall higher proportion of TM CD4+ T cells than observed for blood of the QFT- group (56.8% vs. 44.3%, *P* = 0.0004). Similarly, higher percentages of TTM and TEM cells were detected in CD4+ cell populations of QFT+ subjects as compared to percentages of both cell types in CD4+ cell populations of QFT- subjects (44.1% vs. 36.1% for TTM, respectively, *P* = 0.002; 12.4%vs. 7.7% for TEM, respectively, *P* = 0.0066). By contrast, the proportion of TN T cells of the CD4+ T cell population (50.5%) of the QFT- group exceeded that of the QFT+ group (38.8%, *P* = 0.001) (Fig. 2).

**Fig. 3:**
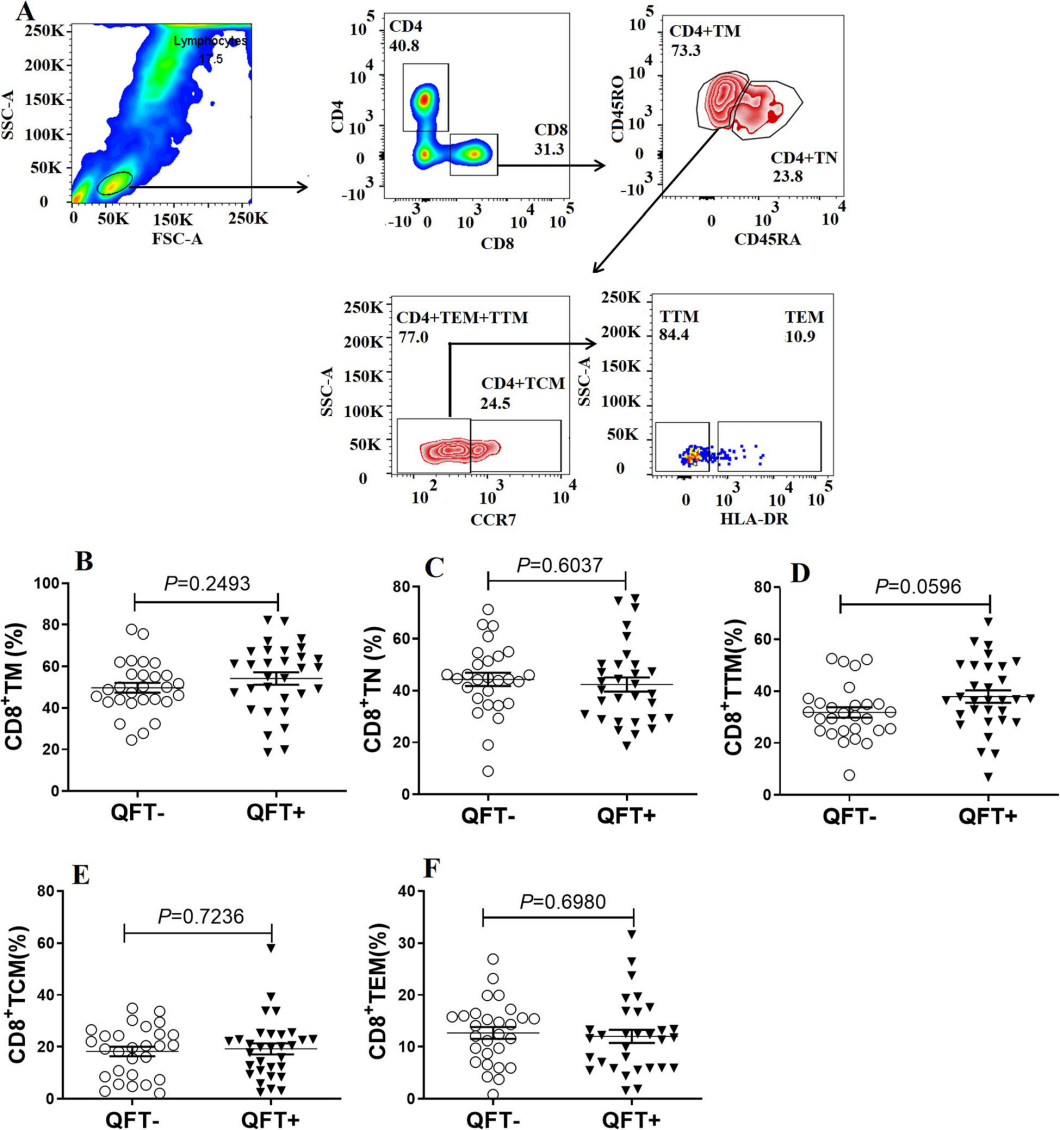
Gating strategy and the expression profiles of memory CD8+ T cell subpopulation in latent TB-infected HCWs. **A** Gating strategies and representative results for the CD8+ T cell subpopulation. **B** Analysis of expression differences between memory CD8+ T cells in QFT+ and QFT- groups. **C**Analysis of expression differences between naive CD8+ T cells in QFT+ and QFT- groups. **D** Analysis of expression differences between transitional memory CD8+ T cells in QFT+ and QFT- groups. **E**Analysis of expression differences between central memory CD8+ T cells in QFT+ and QFT- groups. **F** Analysis of expression differences between effector memory CD8+ T cells in QFT+ and QFT- groups

We also compared CD8+ T cell subpopulations between QFT+ and QFT- groups, with results provided in Fig. 3. Briefly, percentages of various memory CD8+ T cell subpopulations in the QFT+ group were comparable to corresponding QFT- group percentages (*P* > 0.05).

### Comparison of PD-1 expression on peripheral blood CD4+ and CD8+ T cells

**Fig. 4:**
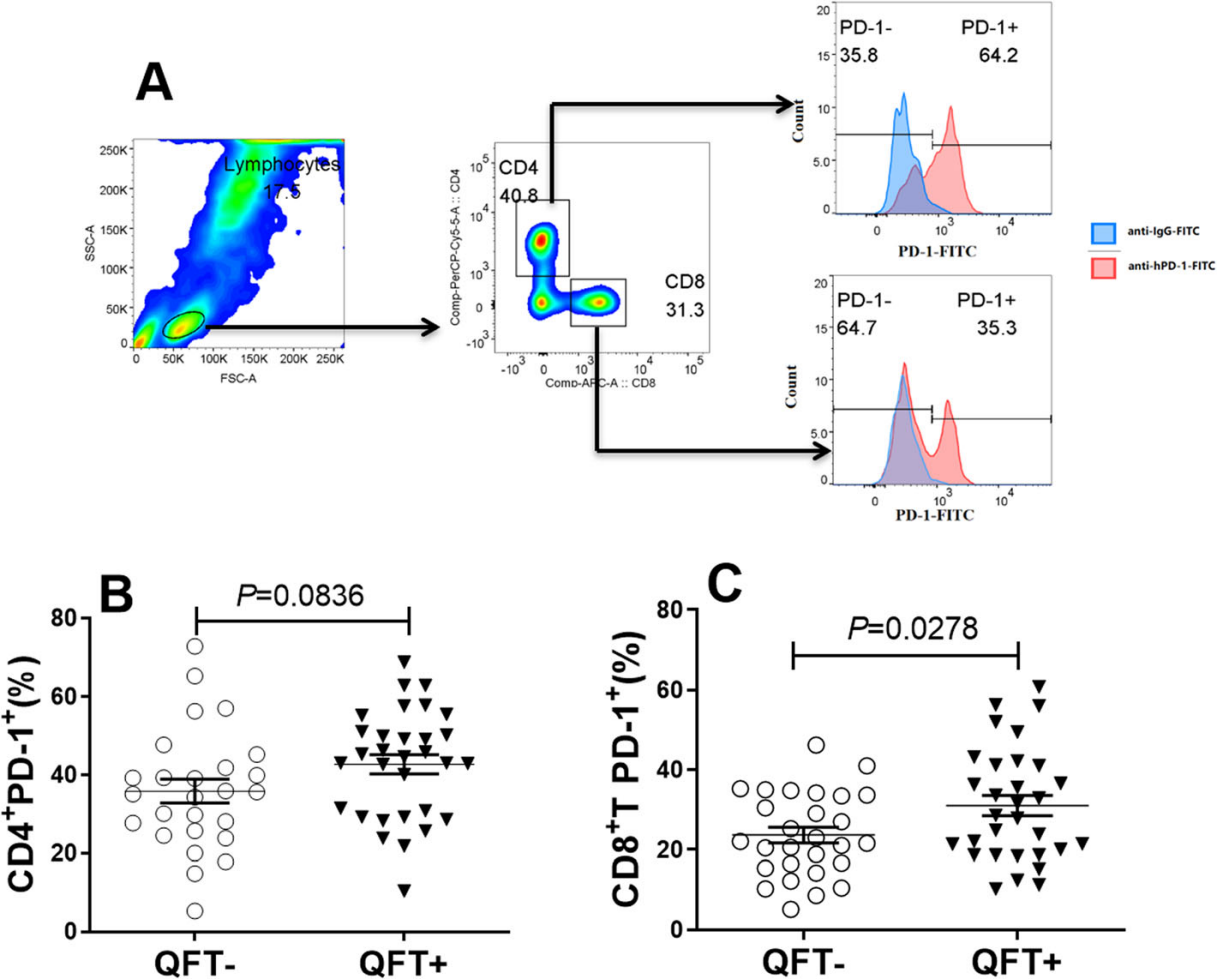
Expression of PD-1 on T cells in latent TB-infected HCWs. **A** Gating strategies and representative results related to PD-1 expression on T cells. **B** Analysis of PD-1 expression differences between CD4+ T cells in QFT+ and QFT- groups. **C** Analysis of PD-1 expression differences between CD8+ T cells in QFT+ and QFT- groups

Using flow cytometry, levels of PD-1 receptors on surfaces of peripheral T lymphocytes were stratified by T cell type (CD4+ versus CD8+). As shown in Fig. 4, the overall percentage of QFT+ group CD8+ T cells with detectible surface PD-1 expression was significantly greater than that of the QFT- group (median, 31.6% vs. 25.3%, *P* = 0.0278). By contrast, no statistical difference in percentage of CD4+ T cells with detectible PD-1 expression was observed between QFT+ and QFT- groups (median, 44.6% vs. 34.3%, *P* = 0.0836). Furthermore, the percentage of all subsets of CD8+ T memory cells with detectible PD-1 expression in the QFT+ group was significantly greater than that of the QFT- group (Fig. S1). Meanwhile, no statistical difference was observed between QFT+ and QFT- groups of percentages of naïve CD4+ T cells with detectible surface PD-1, the main CD4+ T memory cell subset (Fig. S2).

## Discussion

Despite implementation of infection control measures, HCWs remain at high risk of contracting occupational tuberculosis [2]. In this study, our results suggested that nearly one quarter of HCWs in a TB-specialized hospital in China had LTBI, a lower rate than the rate of 39% reported in a systematic review conducted in HCWs from low- and middle-income countries [7, 18]. Of note, a recent population-based cohort study in China revealed that LTBI prevalence was 19% in the rural population, a rate only slightly lower than that found here [18]. There are several explanations for the relatively low rate of LTBI in our report. On the one hand, the prevalence of LTBI generally positively correlates with TB incidence rates across regions. For example, Apriani and colleagues revealed that HCWs from countries with annual TB incidence rates of > 300/100,000 displayed the highest LGBI prevalence rates [7]. Thus, the relatively lower LTBI rate for HCWs found here likely reflects declining TB incidence in China as compared to other countries [19]. On the other hand, the risk of occupational TB infection has been substantially mitigated by implementation of the multi-level infection control approach practices in our hospital, which includes use of N95 masks and negative-pressure patient rooms. Our data confirm that strengthened infection control measures are necessary to reduce risk of TB transmission, especially in high-risk settings where TB patients receive care.

An increased risk of LTBI in healthcare settings has been associated with occupational category, increasing age and length of service [8, 20]. However, only length of service was found to be an independent risk factor for occupational infection in our cohort. This strong association likely reflects increased risk due to prolonged occupational exposure to TB bacilli, especially in the past when infection control measures were less effective. The fact that older workers are at higher risk of contracting TB may also reflect improvements in TB infection control practices in recent years, thereby decreasing infection risk of younger HCWs with few years of service. A future study to monitor TB conversion in HCWs using an Interferon-γ Release Assay (IGRA) will be key to validating our hypothesis.

The interferon gamma release assay (IGRA) is useful for monitoring the establishment and persistence of T cell memory for identification of LTBI cases. Detailed analysis of memory T cell subpopulations of QFT+ subjects in this work demonstrated greater proportions of TM CD4+ cells in that group than in the QFT- group, suggesting that the conversion of naïve memory CD4+ cells to mature memory CD4+ cells requires continuous antigenic stimulation through prolonged occupational exposure. This interpretation was supported by the observation that a significantly higher proportion of CD4+ TEM, but not CD4+ TCM, was found in the QFT+ group. Our findings raise an interesting question regarding the diminished response of memory CD4+ cells in QFT- individuals. Current evidence suggests that establishment of T cell memory can be influenced both by antigen dose as well as by antigen presentation-associated factors [21, 22], although exact mechanisms underlying variable T cell memory remain unclear. Here we hypothesize that macrophages of QFT- HCW subjects may possess greatly enhanced tubercle bacilli-killing activity, thereby leading to weak antigen presentation and subsequently diminished memory T cell proliferation.

Another interesting finding of this study was that immunodepletion of CD8+ T cells was observed in QFT+ individuals. Of note, QFT+ individuals tend to produce more sustained cytokine responses due to high proportions of TM and TEM CD4+ cells that promote recruitment and activation of CD8+ T cells. Therefore, in the face of repeated MTB challenges, homeostatic forces must balance the immunological CD8+ cell response to prevent inflammatory damage [23], while preserving the functionality of CD8+ T cells for combating MTB and other pathogens. Specially, in a report by van Pinxteren and colleagues, depletion of CD8+ T cells in mice during the chronic stage of TB infection resulted in greater bacterial burden, indicating these T cells are important for long-term control of MTB infection [24]. Such findings, together with the results of the current study, strongly suggest that the immunodepletion of CD8+ cells in QFT+ HCWs may increase likelihood of latent TB infection that may progress eventually to active TB disease. Therefore, our results highlight an urgent need to identify the dynamics of HCW T cell subpopulations taking into account variable cell life spans in order to enhance our understanding of immunological mechanisms of human MTB infection.

We also acknowledge several limitations of this study. First, the statistical power of our analysis may be insufficient due to the small sample size in this work of subjects recruited from a single center. Our analysis of risk factors for HCWs with latent TB would greatly benefit from analysis of data drawn from a larger number of subjects to generate results that are statistically more robust. Second, the establishment of immunological memory requires time (several weeks). Thus, the cross-sectional design of the present study may lead to underestimation of LTBI prevalence in our HCW cohort. Finally, despite our observation that lower numbers of CD8+ cells were present in subjects who had experienced repeated MTB exposure, it remains unclear whether MTB-specific CD8+ cells were specifically immunodepleted during repeated MTB exposure.

In conclusion, our data first demonstrated that upregulation of PD-1 expression on circulating CD8+, but not CD4+ T cells, was associated with LTBI of HCWs. Moreover, occupational TB infection risk was substantially mitigated by implementation of a multitiered implementation of infection control measures in our hospital. Only length of service was noted as an independent risk factor for occupational infection. Further study is urgently needed to determine the mechanism of HCW CD8+ immunodepletion in response to repeated MTB exposure.

## Supplementary Information

**Additional file 1: Supplemental Figure 1.** The expression of PD-1 of CD4 + T cell subpopulation in latent TB infection HCWs.

**Additional file 2: Supplemental Figure 2**. The expression of PD-1 of CD8 + T cell subpopulation in latent TB infection HCWs.

## Data Availability

Datasets obtained and/or analyzed during the current study are available from the corresponding author upon request.
