# Supplementary figures and images for: Upregulation of PD-1 expression on circulating CD8+ but not CD4+ T cells is associated with tuberculosis infection in health care workers
Source: BMC Immunol. 2021 Jun 25;22:39. doi: 10.1186/s12865-021-00433-9 (PMC8234730; doi:10.1186/s12865-021-00433-9)

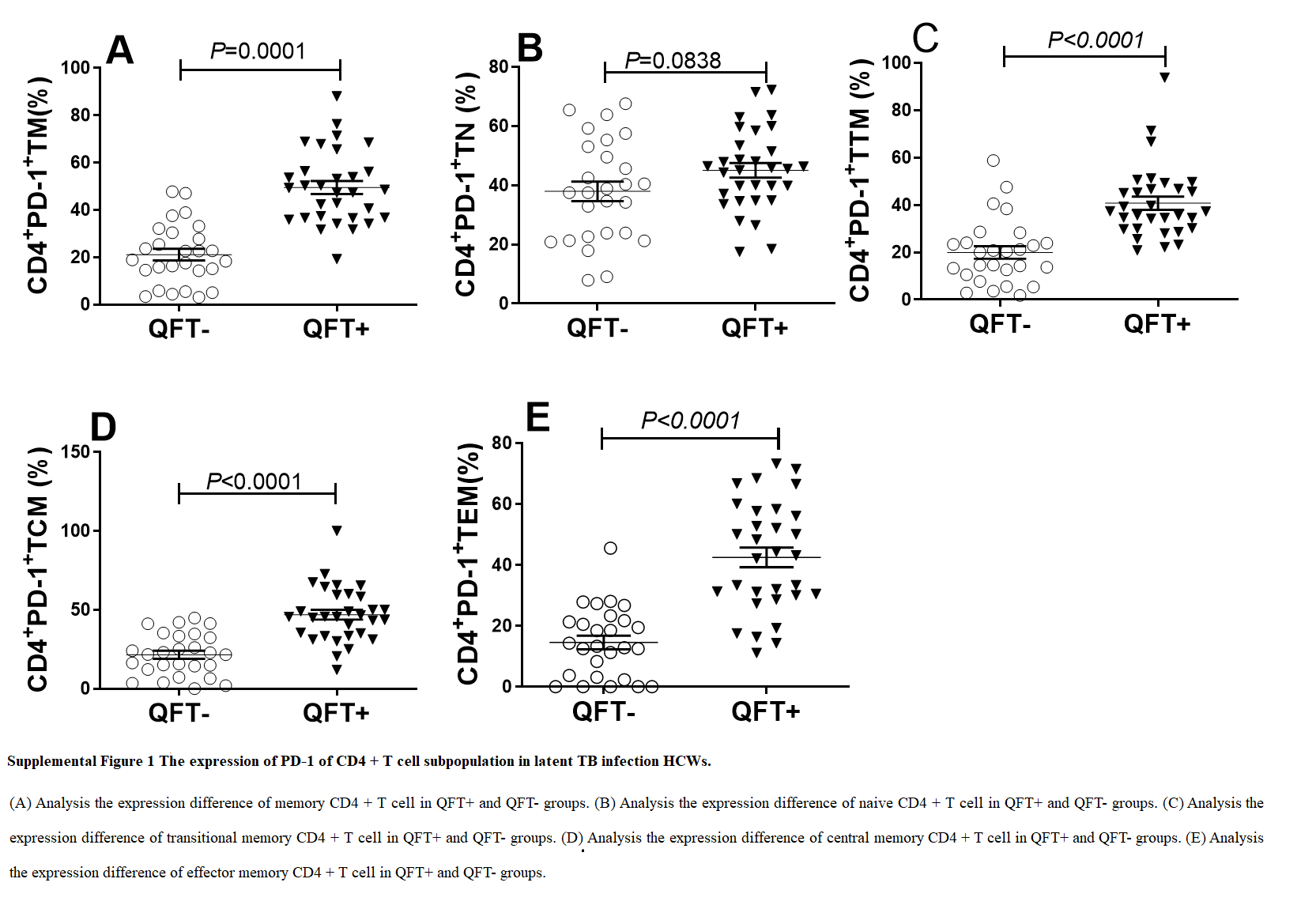

Supplement: Supplementary file 1 — Additional file 1: Supplemental Figure 1. The expression of PD-1 of CD4 + T cell subpopulation in latent TB infection HCWs. [file 12865_2021_433_MOESM1_ESM.tif]

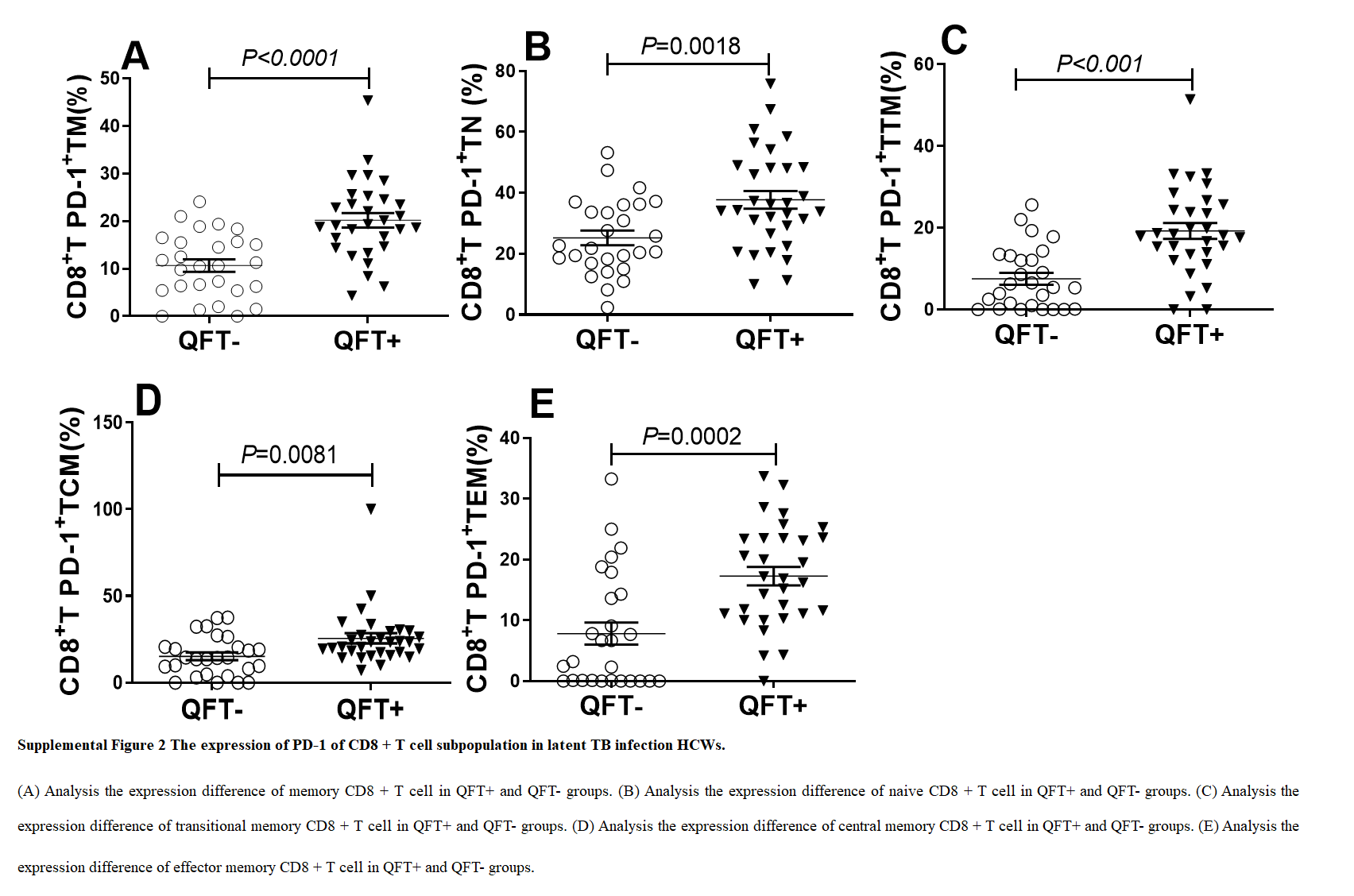

Supplement: Supplementary file 2 — Additional file 2: Supplemental Figure 2. The expression of PD-1 of CD8 + T cell subpopulation in latent TB infection HCWs. [file 12865_2021_433_MOESM2_ESM.tif]
